# Supplementary figures and images for: IDO1 Expression in Ovarian Cancer Induces PD-1 in T Cells via Aryl Hydrocarbon Receptor Activation
Source: Front Immunol. 2021 Apr 16;12:678999. doi: 10.3389/fimmu.2021.678999 (PMC8136272; doi:10.3389/fimmu.2021.678999)

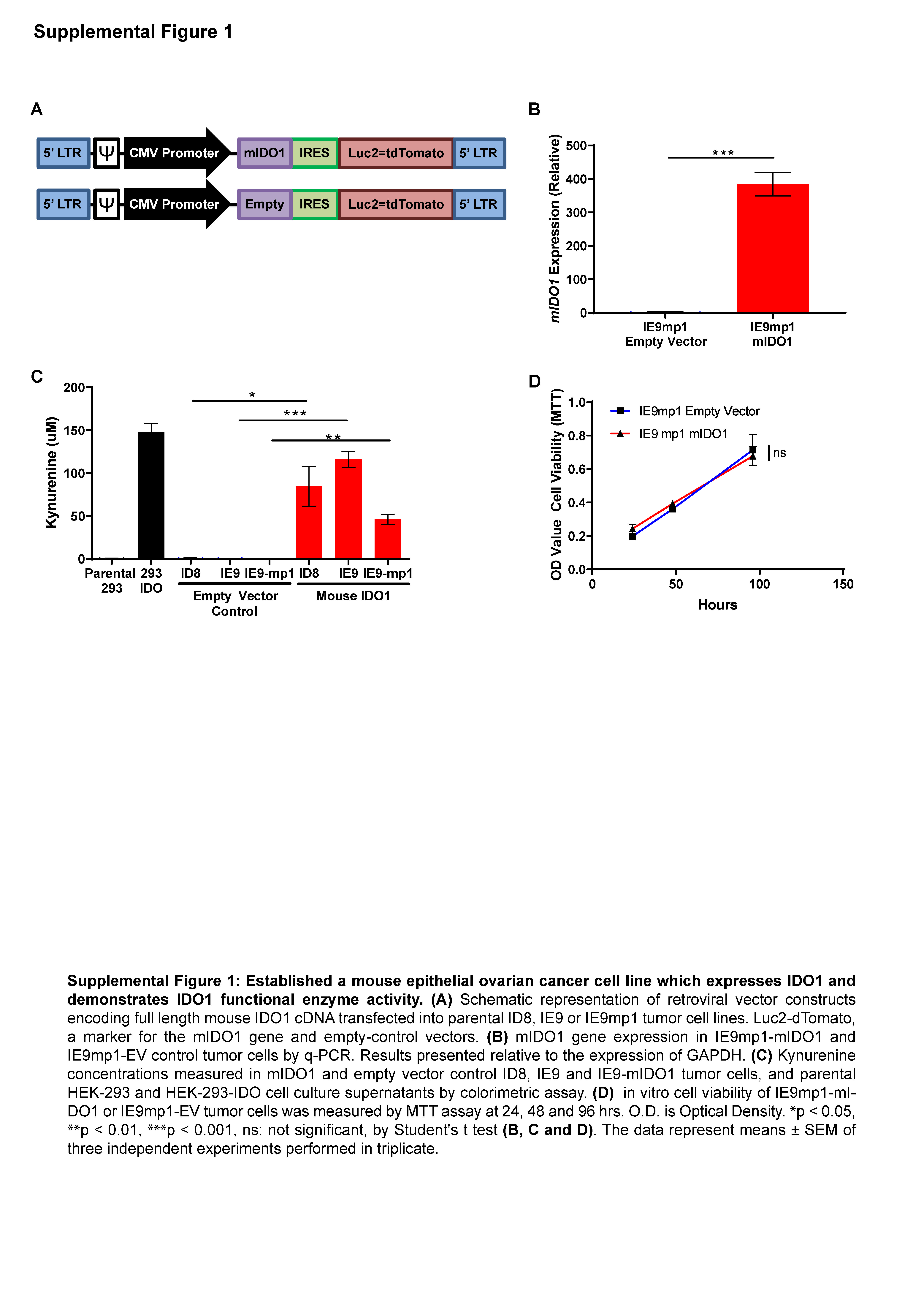

Supplement: Supplementary file 1 [file Image_1.tif]

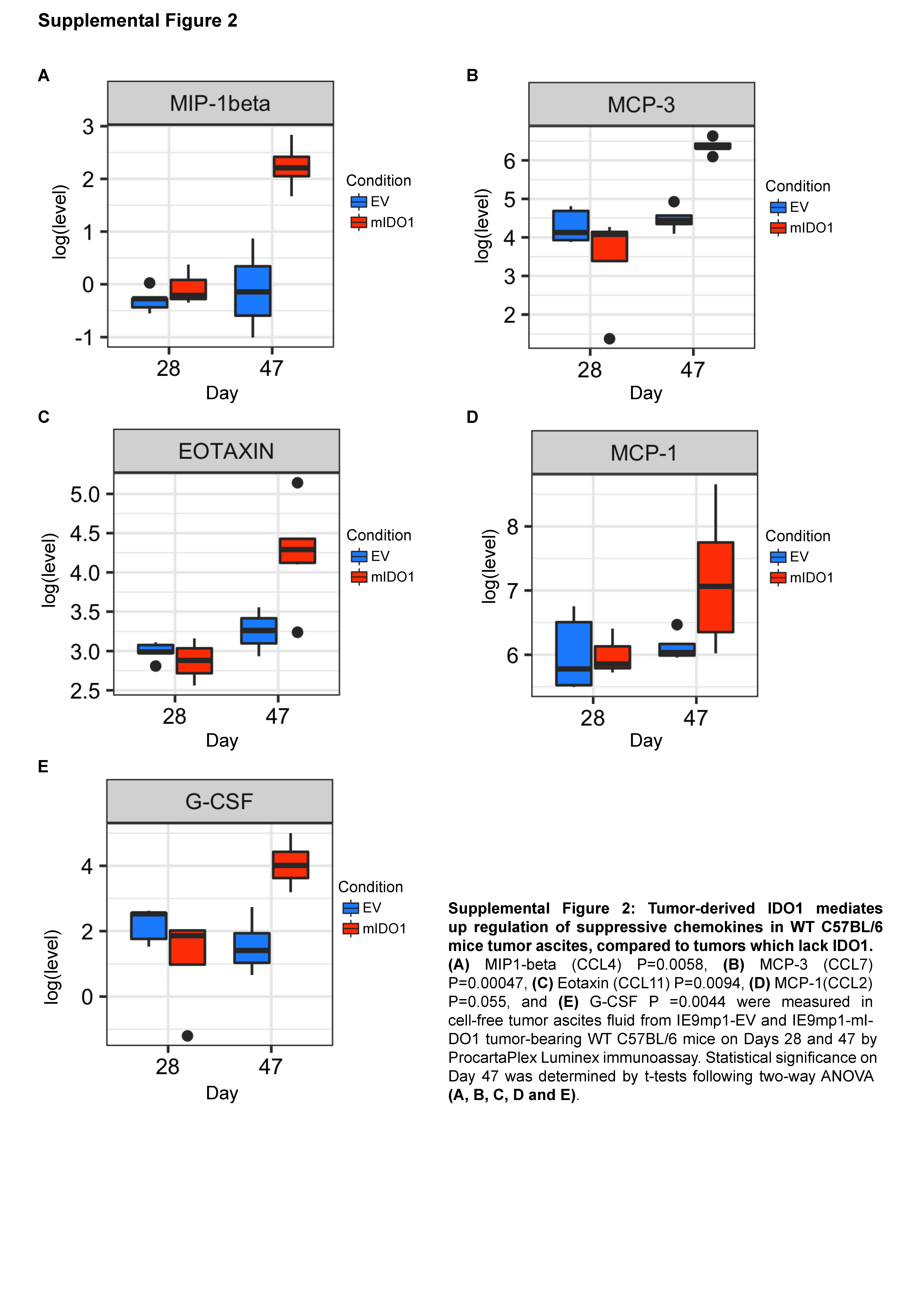

Supplement: Supplementary file 2 [file Image_2.tif]

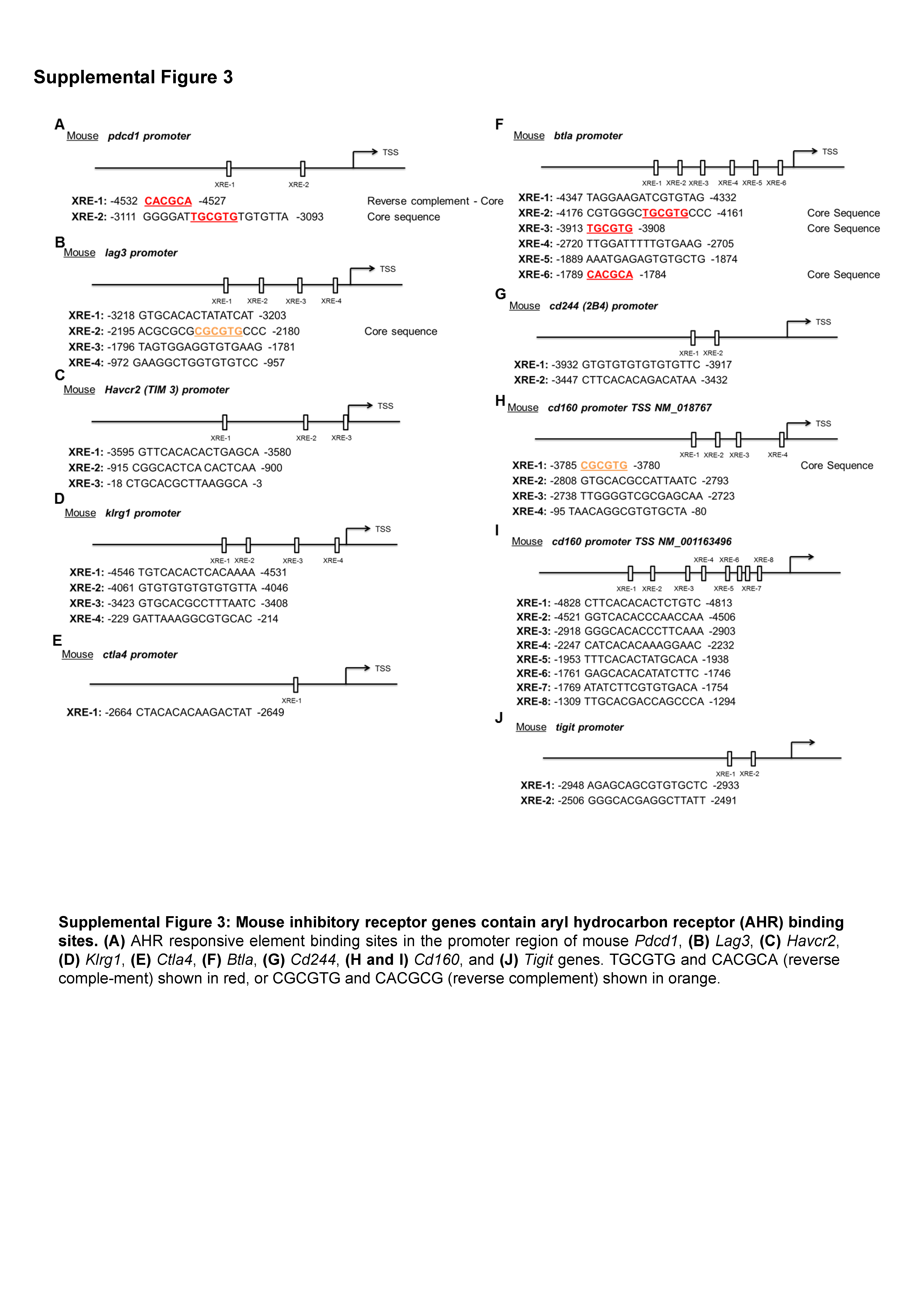

Supplement: Supplementary file 3 [file Image_3.tif]

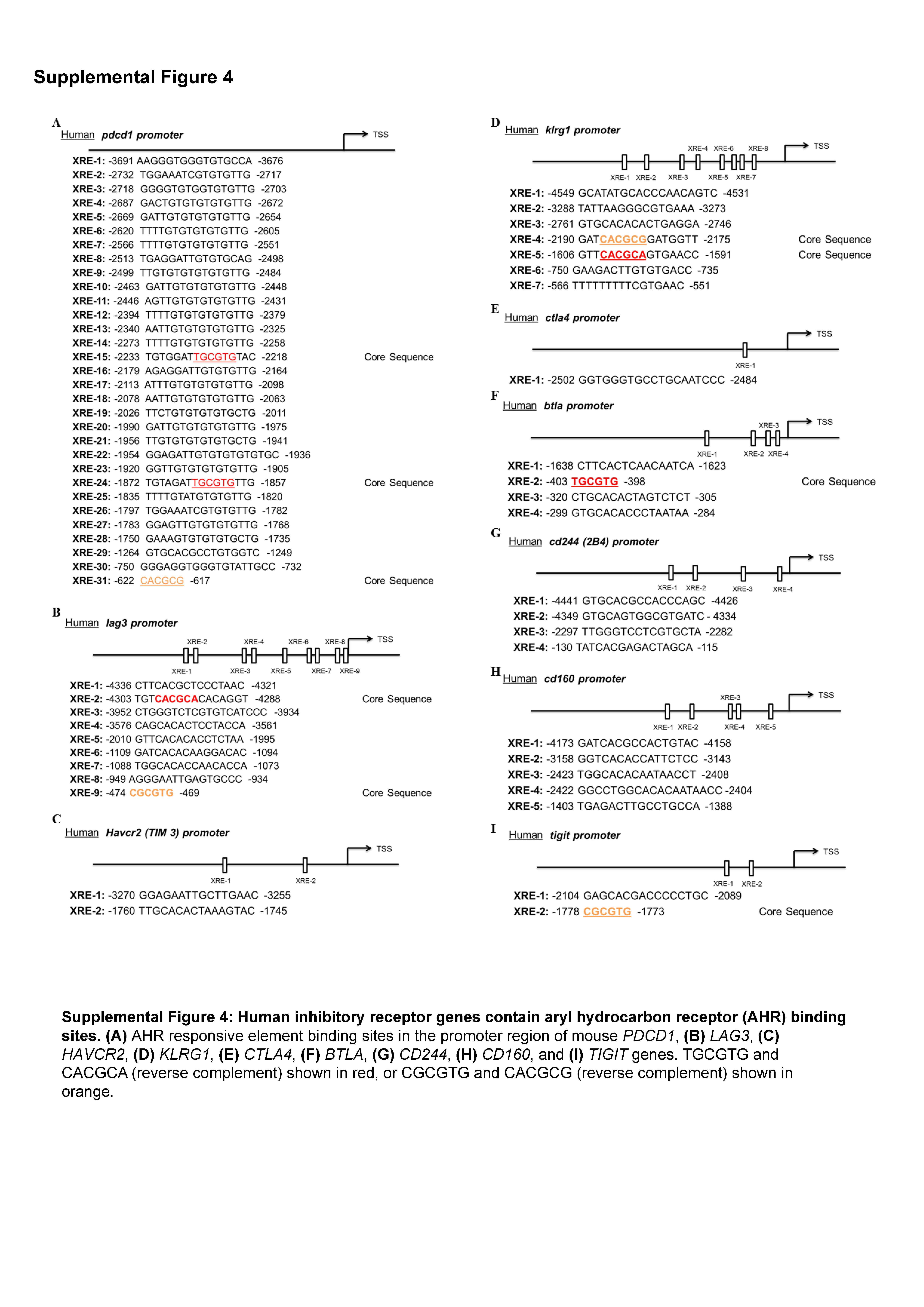

Supplement: Supplementary file 4 [file Image_4.tif]

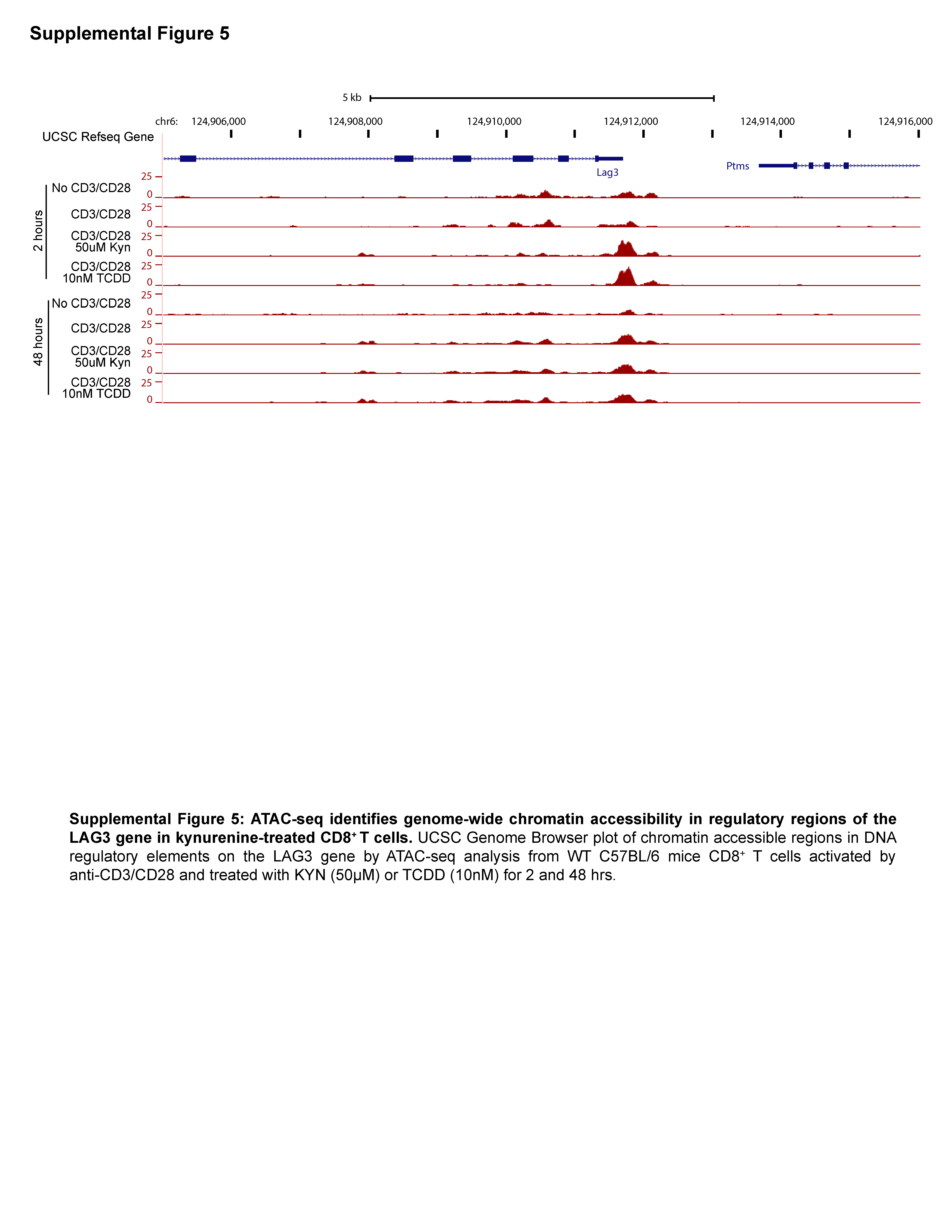

Supplement: Supplementary file 5 [file Image_5.tif]

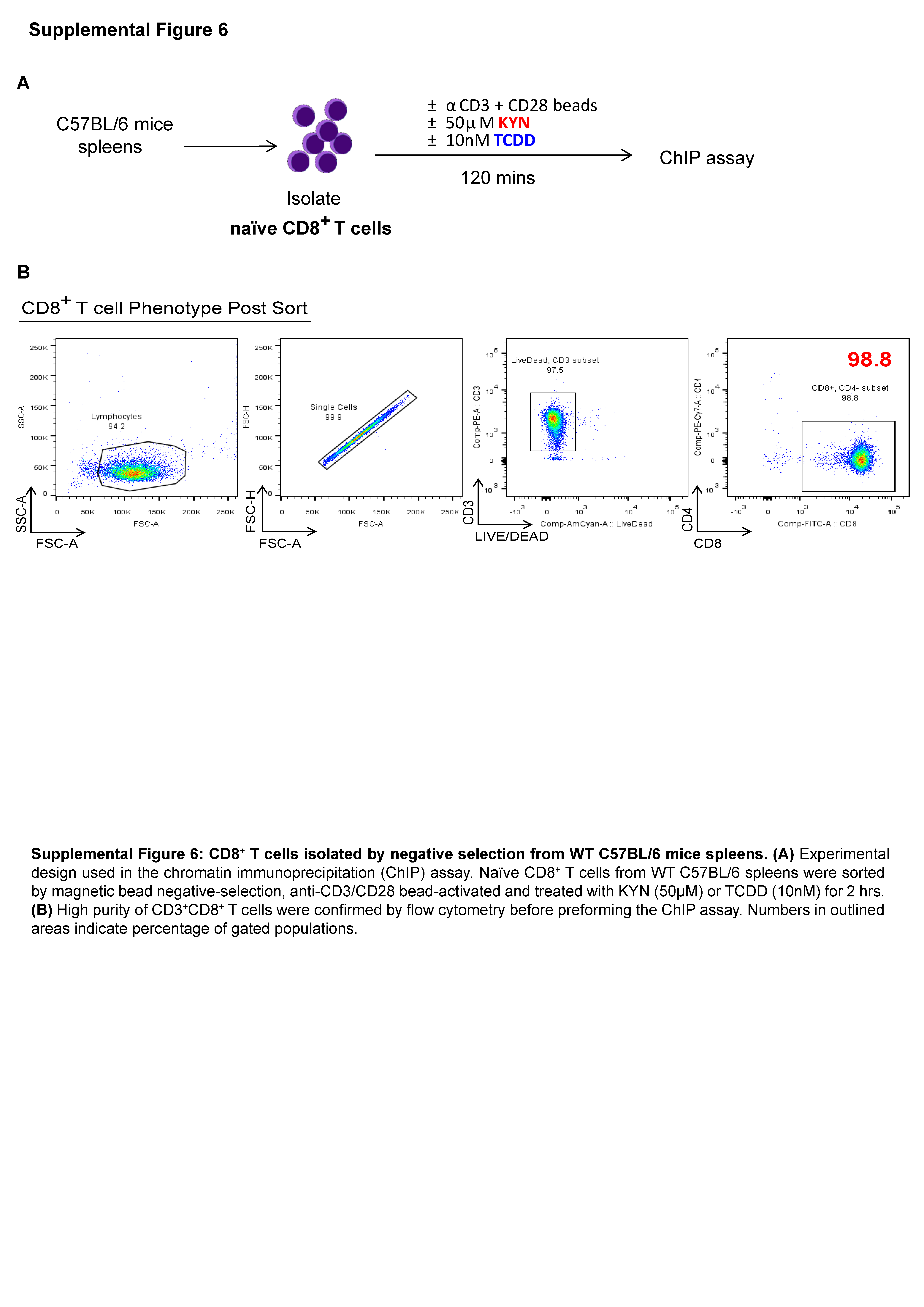

Supplement: Supplementary file 6 [file Image_6.tif]

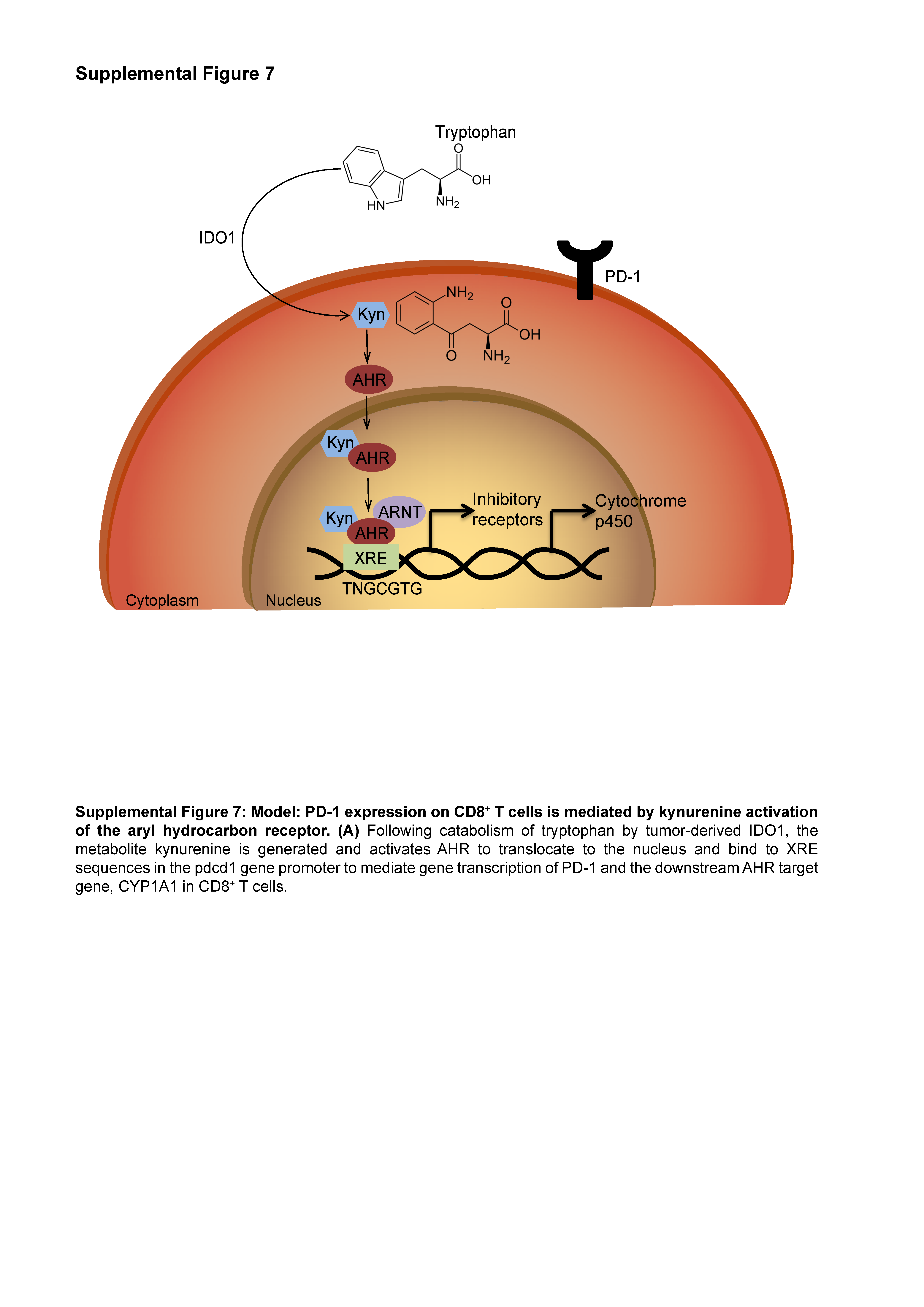

Supplement: Supplementary file 7 [file Image_7.tif]
