# Supplementary material for: IDO1 Expression in Ovarian Cancer Induces PD-1 in T Cells via Aryl Hydrocarbon Receptor Activation
Source: Front Immunol. 2021 Apr 16;12:678999. doi: 10.3389/fimmu.2021.678999 (PMC8136272; doi:10.3389/fimmu.2021.678999)
Supplement: Supplementary file 8 [file Table_1.docx]

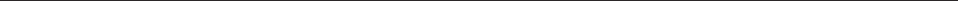

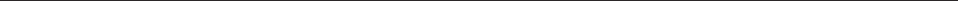

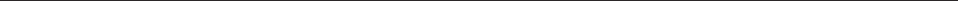

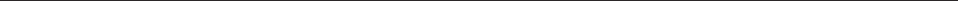

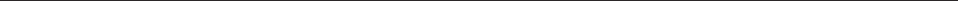

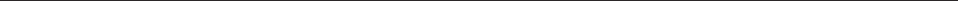

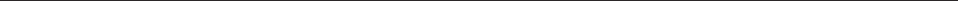

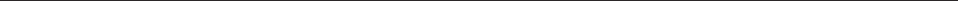

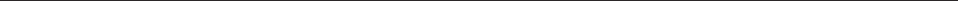

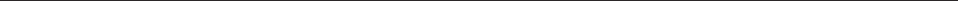

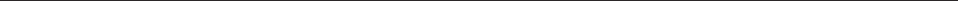

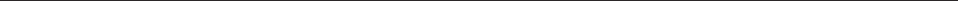

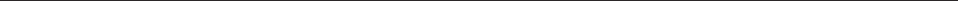

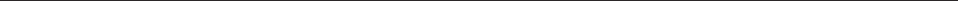

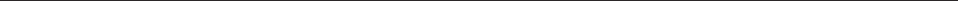

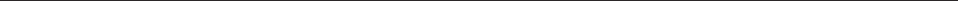

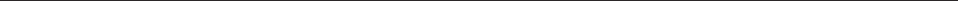

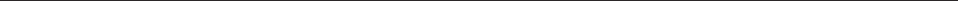

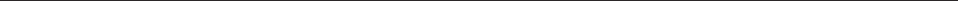

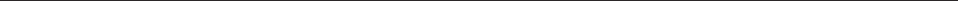

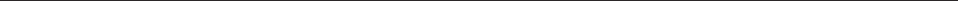


**Supplemental Table 1: Metabolites impacted by IDO1**

**Metabolites**

**Main effect (IDO vs EV)**

**p-value**

**Interaction**

**p-value**

**Difference at Day 47 (IDO vs EV)**

**p-value**

Nicotinamide

0.189032

0.602159

0.223086

0.654903

1.621604

0.151848

Anthranilic acid

1.206492

0.029123

-0.23091

0.738988

2.633593

0.045494

3-Hydroxy- anthranilic acid

1.382396

0.028846

-0.78079

0.332471

1.972503

0.094569

Kynurenine

0.090664

0.722561

-0.06909

0.844123

0.078978

0.939739

Quinolinic acid

0.360513

0.202653

-0.40867

0.289857

-0.20885

0.842954

Tryptophan

-0.01302

0.950294

-0.29373

0.317316

-1.80451

0.122412

Kynurenic acid

0.091745

0.803167

-1.69272

0.004695

-5.30438

0.003879

3-Hydroxy- kynurenine

0.627715

0.163895

-1.11163

0.080462

-1.76866

0.125822

Cytidine mono- phosphate

-3.67827

0.064032

2.74784

0.292589

-0.6193

0.563978

Cytosine

0.996656

0.216081

-1.44731

0.193921

-0.97814

0.390457

Aspartic acid

-0.02678

0.937816

-0.23048

0.627808

-1.09535

0.312779

Uridine mono- phosphate

-0.85974

0.17032

0.819912

0.333562

-0.06233

0.95243

Inosine- mono- phosphate

0.465642

0.366114

0.275987

0.693738

1.799794

0.130044

Adenosine mono- phosphate

0.102399

0.869628

0.764493

0.381058

1.608241

0.163749

De- oxyguanosine mono- phosphate

0.202771

0.768884

0.450871

0.636425

1.142681

0.30149

Guanosine mono phosphate

-0.63244

0.146429

1.407003

0.026943

2.26263

0.058107

Uric acid

-0.01987

0.970336

0.226668

0.75874

0.841109

0.434955

Adenine

0.60252

0.170071

-0.02619

0.964159

1.591071

0.167196

Hypoxanthine

0.739288

0.094298

-0.18501

0.748375

1.471733

0.189358

De- oxyadenosine

1.207014

0.016706

-0.1821

0.76861

2.23376

0.061032


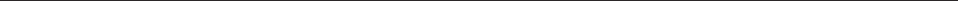

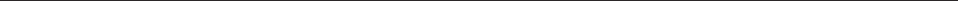

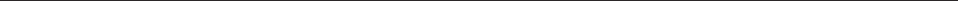

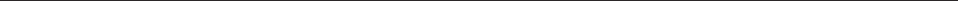

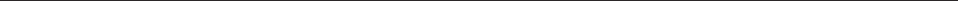

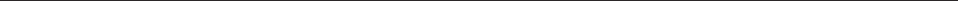

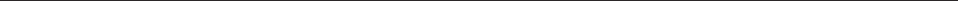

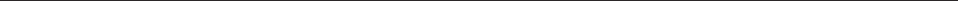

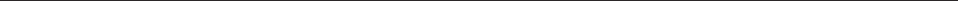

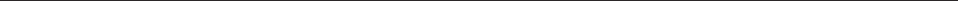

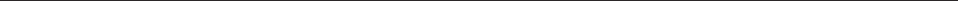


mono- phosphate

Nicotinic acid

0.277439

0.518325

0.365782

0.536408

2.826733

0.03997

Xanthine

1.368548

0.014973

-0.5495

0.42909

2.087495

0.077204

Uridine

0.426486

0.369973

-0.01787

0.977907

0.876607

0.420153

Adenosine

1.239444

0.055597

0.052214

0.949752

3.625779

0.018015

Guanosine

0.713478

0.207114

-0.4344

0.567611

0.599134

0.571715

Inosine

1.483118

0.023859

-0.33553

0.681537

2.163445

0.088951

Guanine

1.070111

0.090418

-0.93002

0.269584

0.283867

0.788158

purine

0.689567

0.134366

-0.52413

0.394627

0.411122

0.695514

NADP

1.77888

0.000935

-2.41841

0.001042

-1.62584

0.148013

Thymine

0.998879

0.022095

-0.22964

0.671907

3.098671

0.017356
